# LL‐37: Biological Mechanisms and Emerging Therapeutic Applications in Intestinal Disease

**DOI:** 10.1002/iid3.70451

**Published:** 2026-05-07

**Authors:** Qichao Liu, Peng Xu, Cheng Zhang

**Affiliations:** ^1^ Department of General Surgery General Hospital of Northern Theater Command Shenyang Liaoning Province China; ^2^ Jinzhou Medical University Jinzhou Liaoning Province China

**Keywords:** antimicrobial peptides, colorectal cancer, Gut microbiota, gut–Immune Axis, Inflammatory bowel disease, LL‐37

## Abstract

Human cathelicidin peptide LL‐37 is encoded by the CAMP gene and plays a key role in innate immunity. It maintains intestinal homeostasis through antibacterial, immunomodulation, and tissue repair functions. This paper reviews the multiple functions of LL‐37 in the intestinal‐immune axis and its contribution to intestinal immune homeostasis. A large amount of evidence shows that the biological effect of LL‐37 is highly dependent on the environmental background, and its effects vary with peptide concentration, receptor binding status, disease stage, and local microenvironment. This article reviews the latest findings of the dual role of LL‐37 in inflammatory bowel disease (IBD) and colorectal cancer (CRC), and focuses on the conditional mechanism of the transformation of its activity from protective to pathogenic. We also discuss the interaction between LL‐37 and intestinal microbiota, focusing on how microbial signals and host peptides can coordinate to regulate mucosal immunity. At the same time, this article examines the key obstacles to the therapeutic application of LL‐37 and its clinical promotion: cytotoxicity, rapid degradation by proteases, and drug resistance. We have further explored new strategies to overcome these challenges in the near future, including peptide engineering, nanocarrier delivery systems, and combined therapy. These findings together position LL‐37 at the intersection of intestinal immunity and microbial ecology, providing a theoretical basis for its therapeutic application in IBD, CRC and infectious colitis.

## Graphical abstract

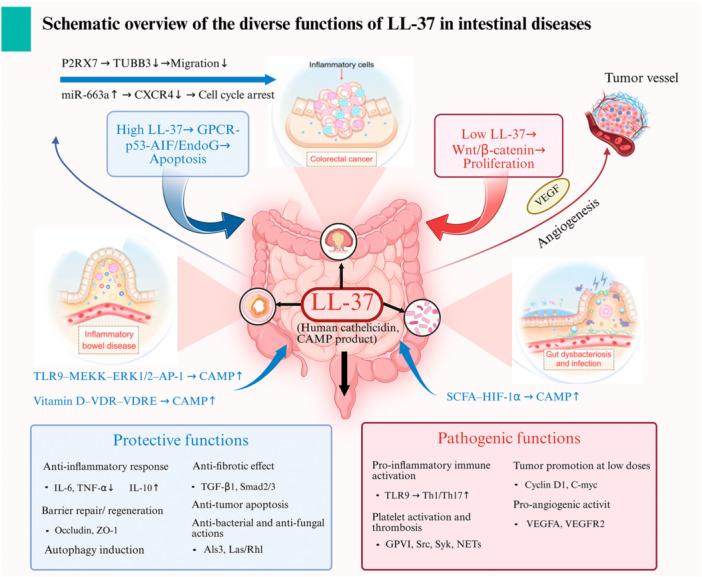

## 1 Introduction

The intestine is a highly dynamic and complex ecosystem composed of intestinal epithelial cells, immune cells, and symbiotic microbial communities. These components coordinate to regulate key physiological functions, including maintaining the integrity of the epithelial barrier, regulating the immune response, and resisting pathogen invasion [1]. The destruction of the intestinal epithelial barrier, accompanied by intestinal microbial dysregulation, has been increasingly recognized as the core driver of the onset of gastrointestinal diseases [2].

Antimicrobial peptides (AMPs) play an important role in maintaining intestinal homeostasis. Among them, human LL‐37 and mouse cathelicidin‐related antimicrobial peptide (CRAMP) are representative members of the cathelicidin peptide family involved in host defense. LL‐37 is the only human cathelicidin peptide encoded by the *CAMP* gene. As a cationic peptide composed of 37 amino acids, it adopts an amphiphilic α‐helical structure. After its precursor protein, hCAP‐18, is cleaved by serine protease 3, LL‐37 is converted into an active form. After processing, LL‐37 is widely expressed in epithelial barrier tissues such as skin, respiratory tract, and gastrointestinal mucosa [3]. Its expression is regulated by a variety of host and microbial signals, including microbial‐related molecular patterns (MAMPs), bacterial metabolites, inflammatory cytokines, and vitamin d‐VDR signaling pathways [4]. In addition to direct antibacterial effects (such as destroying microbial membrane structure and inhibiting biofilm formation), LL‐37 can also regulate inflammatory response and promote tissue repair by regulating immune signaling pathways, such as Toll‐like receptors (TLR), PI3K/AKT, and NF‐κB [5]. The latest research shows that LL‐37 is involved in the pathogenesis of a variety of intestinal diseases [6].

This paper proposes that LL‐37 serves as a context‐dependent regulatory factor for intestinal homeostasis, and its expression changes may affect the balance between inflammation subsidence and disease progression. Although the immunomodulatory characteristics of LL‐37 have been widely reported, its organ‐specific function in the intestinal microenvironment has not been fully clarified. This article reviews the latest research progress on the structural characteristics and regulatory mechanisms of LL‐37 expression, and explores its dual protection and pathogenic role in IBD and CRC. At the same time, the two‐way interaction between LL‐37 and intestinal microbiota is explained, especially focusing on its key role in the intestinal‐immune axis—an aspect that is rarely emphasized in the early antimicrobial peptide reviews. In addition, we explored the emerging paradigm of LL‐37 synergistic microbial metabolites and probiotics to maintain intestinal immune homeostasis. Based on its mechanism framework, we look forward to how the future treatment strategy for intestinal diseases will integrate the characteristics of LL‐37 and discuss it in combination with the latest delivery technology progress.

## 2 Biological Properties of LL‐37

As a key effector molecule of the innate immune system, LL‐37 is widely expressed in a variety of tissues of the human body. When stimulated by pathogens, LL‐37 activates downstream signal cascading reactions by binding with a variety of cell surface receptors, thus promoting *CAMP* gene transcription and enhancing its secretion, thus exerting immunomodulation function [7, 8].

### 2.1 Antibacterial Properties of LL‐37

LL‐37 has broad‐spectrum antibacterial activity against Gram‐positive bacteria (such as *Staphylococcus aureus*) and Gram‐negative pathogens (including *Klebsiella pneumoniae*, *Neisseria gonorrhoeae*, and *Escherichia coli*) [9, 10, 11]. Its sterilization mechanism mainly destroys the anionic components of the bacterial membrane through charge‐dependent interactions, resulting in structural disintegration and cell dissolution [12]. In addition, LL‐37 can produce intracellular effects by binding to macromolecules such as nucleic acids, interfering with the key physiological processes of bacteria [13]. Its prominent feature is that it has strong anti‐biofilm activity. It can prevent the initial adhesion and biofilm formation of pathogenic bacteria such as *Pseudomonas aeruginosa*, and can also destroy the mature biofilm of *Staphylococcus aureus* when used at high doses [12, 14, 15]. Its mechanism of action may involve activating bacterial SOS stress response and interfering with the quorum‐sensing systems [15, 16, 17]. In addition, LL‐37 plays an antibacterial role by neutralizing bacterial endotoxins and inhibiting pro‐inflammatory reactions [18, 19]. For example, LL‐37 inhibits *Helicobacter pylori* colonization and infection by destroying biofilm formation, damaging membrane integrity, and regulating host immune response [20].

### 2.2 Antifungal Properties of LL‐37

LL‐37 has broad‐spectrum antifungal activity, which can inhibit the growth of pathogenic fungi, including *Aspergillus* and *Candida* [21]. Its mechanism of action is not limited to destroying cell membranes: LL‐37 can also penetrate the fungal cell membrane and accumulate within cells, thus inducing the production of reactive oxygen species (ROS), triggering oxidative stress, and ultimately leading to the death of fungal cells [21, 22]. The number of cases of *Aspergillus* infection is increasing, and the resistance to traditional antifungal treatment is increasing. It is urgent to develop alternative interventions. LL‐37 directly acts on the surface of fungal cells and destroys the integrity of the cell wall. In addition, it regulates the host's immune response by inhibiting the release of pro‐inflammatory cytokines, showing the dual role of controlling pathogens and immunomodulation [23, 24]. As a multidrug‐resistant fungal pathogen, *Candida auris* has caused more and more concerns in clinical practice. Studies show that LL‐37 can induce the cell cycle to stagnate in the S phase of DNA synthesis, which leads to programmed cell death [25].

### 2.3 Antiviral Characteristics of LL‐37

LL‐37 has shown strong antiviral effects in a variety of physiological and pathological environments. It can inhibit the replication of a variety of viruses (such as respiratory viruses, *adenoviruses*, *herpes simplex virus*, and HIV) and enhance local immune defense [26, 27]. The substance acts on multiple links of the viral replication cycle: LL‐37 directly attacks the viral envelope or envelope structure to destroy the integrity of the virus [28, 29]; at the same time, it can block the virus from invading the host cell and inhibit virus replication, effectively inhibiting the spread of the virus [30, 31]. Aloul and other studies confirmed that the receptor binding domain (RBD) of LL‐37 and SARS‐CoV‐2 spike protein (S1 subunit) has high‐affinity binding ability, which blocks its binding to ACE2 receptors through the spatial resistance effect, thus effectively inhibiting viral attachment and cell invasion [32]. In addition, LL‐37 can activate the interferon (IFN) signaling pathway by regulating the innate immune response, inducing the expression of interferon‐stimulating genes (ISGs), thereby enhancing the host's antiviral defense ability [29, 33].

### 2.4 Immunomodulatory Activity of LL‐37

LL‐37 shows a wide range of immunomodulatory activities, and its mechanisms of action include recruiting immune cells, promoting angiogenesis, accelerating wound healing, and regulating key processes such as inflammation and apoptosis [34]. Zhai and other studies have shown that LL‐37 activates the MAPK signaling pathway by binding to formyl peptide receptor 2 (FPR2), thus inducing the release of chemokines such as IL‐8, thereby promoting neutrophil collection and enhancing innate immune response [35]. Lee and others reported that LL‐37 accelerates the progression of the disease by activating the inflammatory body and inducing the release of angiogenic mediators, promoting the inflammatory signal transmission and vascular remodeling of rosacea [36]. LL‐37 binding to extracellular DNA can destroy the stability of neutrophil extracellular traps (NETs) and activate autophagy‐related genes in macrophages, thus enhancing the lysosomal degradation of NETs. This synergistic mechanism helps to remove NETs, inhibit the release of pro‐inflammatory cytokines, and alleviate microthrombotic events and out‐of‐control inflammatory cascades [32, 37, 38, 39].

### 2.5 The Effects of LL‐37 on Tumorigenesis

LL‐37 has dual functions in tumor development, and its biological effects depend on the tumor microenvironment and the type of cancer. At present, most studies on LL‐37 focus on its differential expression in cancer, but its potential mechanism is not clear. LL‐37 is highly expressed in lung cancer, breast cancer, and melanoma, which is associated with tumor progression and aggressiveness enhancement; while it shows anti‐tumor activity in gastric cancer and colorectal cancer, highlighting its context‐dependent regulatory effect in different tumor microenvironments [19]. The latest evidence shows that LL‐37 often shows upregulated expression in non‐small cell lung cancer (NSCLC), and its high expression is significantly associated with poor clinical prognosis [40]. Immunohistochemical data confirmed that the increase in LL‐37 expression was associated with the progression of melanoma T staging [41]. On the contrary, LL‐37 inhibits gastric cancer cell proliferation by activating the bone morphogenetic protein (BMP) signal cascade reaction, which can upregulate the cell cycle‐dependent kinase inhibitor p21 and block the cell cycle [42]. The function of LL‐37 in other types of tumors still needs to be further explored.

### 2.6 The Effects of LL‐37 on Other Autoimmune Diseases

More and more studies have confirmed that LL‐37 is a key effective molecule in the pathogenesis and regulation of a variety of systemic diseases. As confirmed by Moreno‐Angarita and others, LL‐37 forms a complex with its own DNA, and is then ingested by plasma cell‐like dendritic cells (pDCs) through the surface Fcγ receptor. This process induces the strong secretion of type I interferon (IFN‐I) and further upregulates the expression of LL‐37, thus promoting the pathogenesis of systemic lupus erythematosus [43]. In addition, LL‐37 can activate P2X7 receptors in keratinizing cells, promote autophagy, enhance the integrity of close connections, and thus enhance the epidermal barrier function. This pathway provides potential treatment targets for self‐inflammatory skin diseases (including psoriasis and atopic dermatitis) [44, 45, 46, 47]. In cardiovascular pathology, LL‐37 activates the TLR4 signal axis to induce the inflammatory activation of coronary endothelial cells in patients with Kawasaki disease (KD), revealing a new molecular target for KD treatment intervention [48].

**Figure 1:**
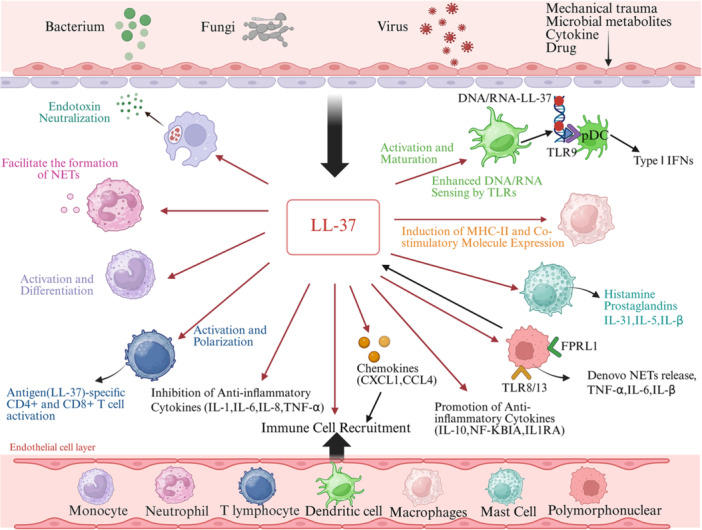
Biological activities of LL‐37.

In summary, as shown in Figure 1, LL‐37 integrates multiple functions through multiple receptor‐mediated pathways, including antibacterial and anti‐biofilm activity, antiviral defense, immunomodulated signal conduction, and tumor regulation. Clarifying its mechanism of action may open up new ways for the treatment, intervention, and clinical management of infectious diseases, chronic inflammatory diseases, and malignant tumors.

## 3 The Role of LL‐37 in Intestinal Diseases

This review explores the association between LL‐37 and a variety of important intestinal diseases, including IBD, CRC, microbiome‐immune axis disorder, and intestinal flora disorder. The following will focus on the mechanism by which LL‐37 plays a protective or pathogenic role in these diseases by participating in specific signaling pathways, receptor interactions, and stage effects. Table 1 summarizes the main experimental and clinical findings of LL‐37 in the fields of intestinal inflammation, infection, and colorectal cancer.

**Table 1:** Representative experimental and clinical studies on the role of LL‐37 in intestinal inflammation, infection, and colorectal cancer.

Author (year)	Study type	Model/Subjects	Research focus	Main findings and conclusions
Hing et al. (2013)	Experimental	Murine models; human colonic epithelial cells	The anti‐inflammatory effect of LL‐37/mCRAMP in *Clostridium difficile* infection and colitis induced by toxin A	Exogenous LL‐37/mCRAMP reduces tissue damage, cell apoptosis, MPO, and TNF‐α levels by inhibiting NF‐κB activation, highlighting its potential as a therapeutic anti‐inflammatory peptide.
Kusaka et al. (2018)	Experimental	Colonic mucosal biopsies from IBD patients (SEMFs)	Expression and modulatory role of LL‐37 in IBD.	LL‐37 was markedly elevated in SEMFs and suppressed LPS‐induced cytokine release, suggesting combined antimicrobial and immunomodulatory activity in IBD.
Koon et al. (2011)	Experimental	Mouse model; human monocytes	Expression, Mechanistic Role, and TLR9‐Dependent Regulation of mCRAMP/LL‐37 in Colitis.	Bacterial DNA activated TLR9 signaling to induce LL‐37/mCRAMP expression, attenuating inflammation and promoting mucosal protection.
Gubatan et al.(2020)	Experimental	Human UC tissues; mouse model	Regulation of LL‐37 by vitamin D in ulcerative colitis.	Elevated vitamin D levels correlated with increased LL‐37 expression and improved mucosal healing, linking vitamin D signaling to intestinal immunity.
Duan et al. (2018)	Experimental	UC patient plasma and tissues; mouse model	Pathogenic role of the LL‐37–bacterial DNA complex in UC.	LL‐37–DNA complexes evaded immune clearance, activated TLR9, and disrupted barrier integrity, thereby exacerbating inflammation and disease progression.
Jiang et al. (2024)	Experimental	Mouse model	Protective effects of CRAMP in DSS‐induced colitis.	CRAMP enhanced epithelial integrity (upregulated occludin, E‐cadherin) and reduced oxidative stress, modulating the microbiota–metabolic axis to mitigate colitis.
Yoo et al. (2015)	Experimental	Mouse model; human colonic fibroblasts	Anti‐fibrotic mechanisms of LL‐37/mCRAMP in colonic fibrosis.	LL‐37 reduced collagen deposition by activating ERK signaling and suppressing TGF‐β1–induced collagen synthesis, alleviating IBD‐associated fibrosis.
Tran et al. (2017)	Clinical cohort	Healthy controls, UC and CD patients	Clinical relevance of serum LL‐37 in IBD.	Serum LL‐37 levels inversely correlated with PMS (UC) and HBI (CD), predicting remission and serving as a noninvasive biomarker for disease activity.
Pircher et al. (2018)	Experimental	Mouse model; human blood and tissue samples	Roles of LL‐37/CRAMP in thrombosis and inflammation.	LL‐37/CRAMP activated platelets and promoted NET formation, aggravating thrombosis and inflammatory responses.
Kuroda et al. (2017)	Experimental	Mouse model; human CRC cell lines	Effects of LL‐37 and its analog FF/CAP18 on colorectal cancer growth.	LL‐37 and FF/CAP18 upregulated miR‐663a and induced G2/M cell‐cycle arrest, suppressing colorectal cancer proliferation.
Wang et al. (2019)	Experimental	Mouse model; human CRC cell lines	Mechanisms of LL‐37‐mediated inhibition of colorectal cancer metastasis.	LL‐37 disrupted microtubule architecture and downregulated TUBB3 expression, suppressing CRC metastatic potential.
Porter et al. (2021)	Clinical cohort	Human CRC tissues and matched normal mucosa	LL‐37 expression and stromal CD8 + T cell infiltrate in CRC.	Epithelial LL‐37 expression intensity decreases with CRC progression but is strongly associated with CD8 + T cell infiltration, suggesting a role in immune surveillance.
Cheng et al. (2015)	Experimental	Mouse models; human colon cancer cells and fibroblasts	Anti‐tumor mechanisms of cathelicidin via CAF and EMT inhibition.	Cathelicidin suppresses colon cancer development by inhibiting the proliferation of cancer‐associated fibroblasts and disrupting EMT.
Fan et al. (2015)	Experimental	Mouse model; intestinal symbionts; HT‐29 cells	Investigating the potential mechanisms by which LL‐37/CRAMP suppresses *Candida albicans* colonization in the gastrointestinal tract.	LL‐37 deficiency increased fungal colonization, indicating its role in maintaining mucosal homeostasis and antifungal defense.
Zhang et al. (2023)	Experimental	Mouse model; vancomycin‐resistant Enterococcus (VRE)	Synergistic bactericidal activity of *lysin* and LL‐37 against MDR *Enterococcus*.	Combined *lysin* and LL‐37 treatment enhanced bacterial lysis and biofilm clearance, suggesting potential as an alternative to antibiotics.
Geng et al. (2025)	Experimental	Mouse models; intratumoral microbiota	CAF‐targeted LL‐37 production for depleting intratumoral pathogens.	CAFs modified by optogenetic techniques can produce LL‐37 in situ, effectively eliminating pathogens (such as *Fusobacterium nucleatum*) within the tumor, and remodeling the tumor microenvironment to inhibit breast cancer metastasis, without interfering with the systemic microbiota.

Abbreviations: CAF, cancer‐associated fibroblast; CD, Crohn's disease; CRC, colorectal cancer; DSS, dextran sulfate sodium; EMT, epithelial‐mesenchymal transition; HBI, Harvey‐Bradshaw Index; IBD, inflammatory bowel disease; MDR, multidrug‐resistant; MPO, myeloperoxidase; NET, neutrophil extracellular trap; PMS, partial Mayo score; SEMFs, superficial erosive mucosal forms; TME, tumor microenvironment; TNF‐α, tumor necrosis factor‐alpha; UC, ulcerative colitis.

### 3.1 The Role of LL‐37 in Inflammatory Bowel Diseases

Ulcerative colitis (UC) and Crohn's disease (CD) are chronic recurrent inflammatory bowel diseases (IBD), which are characterized by persistent inflammation of the gastrointestinal tract [49]. IBD originates from the multi‐dimensional interaction between impaired epithelial barrier function, changes in intestinal microbial composition, and abnormal mucosal immune response [50]. LL‐37 has received increasing attention in IBD research because of its key role in innate immune defense [51]. The peptide is widely expressed throughout the intestine, especially in intestinal epithelial cells, goblet cells, and invasive immune cells. However, its expression patterns vary in different cell types and disease states [52, 53]. Immunohistochemical research shows that in IBD patients, LL‐37 is mainly distributed in the cryptous epithelium of the inflammatory area, while in healthy individuals, it is more evenly distributed in the mucosal layer [54, 55]. Existing evidence shows that LL‐37 has a dual role in the pathogenesis of IBD: it can not only exert a protective effect but also induce pathogenic effects. These effects seem to depend on many variables, including peptide concentration, receptor binding, disease stage, and local inflammatory microenvironment. An in‐depth understanding of the impact of these conditions on the activity of LL‐37 may provide a basis for the development of more accurate treatment strategies.

#### 3.1.1 The Function and Related Mechanism of LL‐37 in Ulcerative Colitis

In UC, LL‐37 can play both protective and anti‐inflammatory effects, and its effect depends on the signaling pathway involved and the molecular morphology of the peptide. This dual function reflects the mechanism by which LL‐37 transforms from a protective mediator to an inflammatory driver under certain conditions.

**Figure 2:**
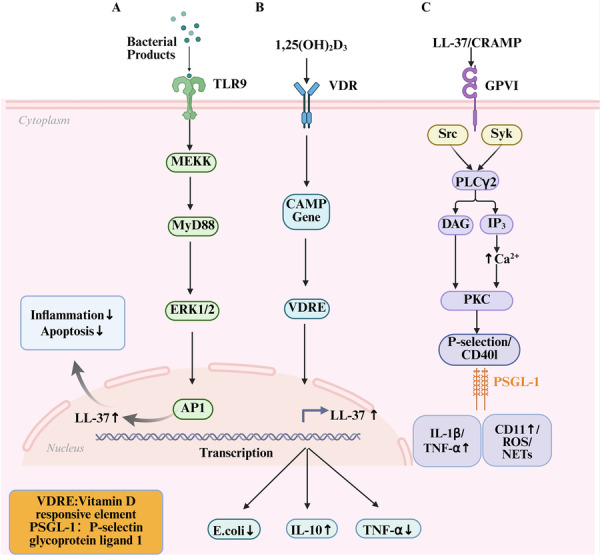
Signaling Axis Governing LL‐37 Induction and Immunomodulatory Activity in the Intestinal Microenvironment. (A) TLR9–MEKK–ERK1/2 Pathway Induces LL‐37 via AP‐1 to Suppress Inflammation and Apoptosis. (B) Vitamin D–VDR Signaling Induces *CAMP* Transcription Through VDRE‐Mediated LL‐37 Upregulation. (C) LL‐37/CRAMP Activates GPVI–PLCγ2–PKC Cascade, Driving Pro‐inflammatory and Thrombotic Responses.

The protective effect of LL‐37 in UC is mainly mediated by receptor‐dependent pathways. Its expression is dually regulated by the TLR signaling pathway and the vitamin D pathway, and the two work together to enhance its anti‐inflammatory and immunomodulatory functions. Koon et al. show that LL‐37 can induce expression through the TLR9‐ERK signaling pathway, thus strengthening the intestinal epithelial barrier function, reducing epithelial cell apoptosis, and alleviating intestinal inflammation (Figure 2A) [55]. Gubatan and others further confirmed that activating the vitamin D signal axis can promote the production of IL‐10 and inhibit pro‐inflammatory cytokines, thus playing an immunosuppressive role in ulcerative colitis (Figure 2B) [56]. In summary, these pathways constitute the main mechanism of LL‐37 to protect the colon mucosa. However, different microenvironments may lead to the opposite effect. Duan and other studies have shown that LL‐37 binds to bacterial DNA (bacDNA) to form an immune complex, which can circulate to the whole body and escape immune clearance, thus weakening the antibacterial activity of LL‐37. These complexes can also activate the TLR9 signaling pathway, promote the polarization of inflammatory T cell subgroups, and ultimately aggravate mucosal inflammation of ulcerative colitis [57]. Therefore, the TLR9 signaling pathway can be protective or pathogenic, depending on whether LL‐37 exists in the form of a free peptide or a complex bound to DNA.

Intestinal microbiota play a key role in regulating the activity of LL‐37. LL‐37 can strongly inhibit the growth of ulcerative colitis‐related pathogens and maintain the integrity of the epithelial barrier. Its mouse homolog, CRAMP, exerts direct bactericidal and anti‐inflammatory effects by interfering with bacterial membrane structure and biofilm formation, which can remove pathogens such as *Escherichia coli* and *Clostridium difficile* [4, 58, 59]. On the other hand, flora disorder may aggravate inflammation and promote the development of the disease [60]. The mucin‐degrading bacterium *Akkermansia muciniphila* (phylum *Verrucomicrobia*) has been shown to improve intestinal barrier function and reduce inflammation. Clinical trials have shown that the abundance of *Verrucomicrobia* in patients with active ulcerative colitis is significantly lower than that of healthy people, and treatment based on intestinal microbiota regulation can not only restore the abundance of *Verrucomicrobia*, but also reduce the content of pro‐inflammatory bacteria (such as *Roseburia*) [61, 62]. Therefore, LL‐37 reduces intestinal inflammation and restores microbial homeostasis by regulating host‐microbiota interaction, inhibiting the excessive proliferation of pathogenic bacteria, and promoting the colonization of beneficial symbiotic bacteria.

The disease stage significantly affects the function of LL‐37. Kusaka et al. found that the expression of LL‐37 in the colon mucosa of patients with ulcerative colitis was significantly upregulated, which alleviated intestinal inflammation by neutralizing lipopolysaccharides (LPS) and inhibiting the release of pro‐inflammatory cytokines [63]. The latest research shows that LL‐37 can adjust the integrity of the epithelial barrier and reduce oxidative stress, thus exerting the function of tissue protection. In the colitis model induced by sodium glucan sulfate (DSS), LL‐37 promotes the expression and structural assembly of closely connected proteins, strengthens the integrity of the epithelial barrier, and restricts the transport of microorganisms and endotoxins through the mucosa [62]. In addition, LL‐37 can remove reactive oxygen or activate antioxidant pathways, reduce the damage of oxidative stress to intestinal tissue, and indirectly protect the colonic mucosal structure [64, 65]. However, in the process of chronic inflammation, persistent barrier dysfunction leads to the accumulation of the LL‐37‐bacDNA complex, which avoids immune clearance, which not only reduces the antibacterial effectiveness of free LL‐37 but also activates the systemic TLR9 signaling pathway to maintain inflammation [57].

#### 3.1.2 The Function and Related Mechanism of LL‐37 in Crohn's Disease

Crohn's disease (CD) is a chronic inflammatory disease that mainly affects the ileum, which can lead to intestinal stenosis, fistula formation, obstruction, and malignant transformation [66]. Compared with healthy people, the transcript and protein expression of LL‐37 in the colon mucosa of patients with active ulcerative colitis were significantly higher. On the contrary, regardless of the degree of inflammatory activity, the LL‐37 levels in the ileal mucosa of patients with Crohn's disease remain relatively stable on average [56]. This difference in anatomical distribution and disease stage shows that the biological activity of the protein is highly affected by the local microenvironment.

LL‐37 plays a complex role in Crohn's disease. It can be used as an anti‐inflammatory and anti‐fibrosis mediator, but it may also promote inflammatory reactions under certain conditions. In its protective effect, LL‐37 plays a role by activating the NOD2 signaling pathway and the intracellular autophagy mechanism, especially when there are NOD2/CARD15 mutations (one of the most important genetic risk factors for Crohn's disease). These functional mutations will destroy autophagy [67]. These mutations interfere with the autophagy process regulated by the intracellular LL‐37 level, which is a key link in congenital immunity, indicating that LL‐37‐dependent autophagy dysfunction is a key mechanism for the occurrence of the disease [44, 68, 69]. Raftery and others confirmed that LL‐37 can enhance autophagy activity and activate the NOD2 signaling pathway, thus alleviating the pathological process associated with Crohn's disease [70]. In addition, in the TNBS‐induced chronic colitis and Salmonella infection model, LL‐37 significantly reduced collagen deposition, reduced fibrosis score, and inhibited collagen mRNA expression. It is more noteworthy that LL‐37 shows a similar anti‐fibrosis effect in human colonic fibroblasts and does not induce abnormal proliferation or apoptosis [71, 72]. Strong evidence from in vivo and in vitro models supports the anti‐fibrosis effect of LL‐37/CRAMP. On the contrary, LL‐37 may aggravate intestinal inflammation by promoting platelet activation during the pathogenic process. As shown by Pircher et al. in Figure 2C, LL‐37/cramp activates platelets through the glycoprotein VI‐dependent Src/Syk signaling axis, thereby inducing the secretion of pro‐inflammatory mediators (such as interleukin‐1β (IL‐1β)). The key mechanism lies in the interaction between platelets and neutrophils. Activated platelets adhere to neutrophils through P‐selectin glycoprotein ligand‐1 (PSGL‐1), thereby activating neutrophils, producing ROS, and forming neutrophil extracellular traps (NETs). These events exacerbate inflammatory reactions and promote arterial thrombosis (Figure 2C) [73]. The biological effect of LL‐37 in Crohn's disease is regulated by receptor specificity: the combination with the NOD2‐autophagy pathway can provide mucosal protection, while the combination with GPVI triggers the unique thrombosis and inflammation‐promoting functions.

Although the dose‐response research on CD is insufficient, clinical evidence shows that circulating LL‐37 levels are closely related to the outcome of the disease. High LL‐37 serum levels are associated with a variety of clinical characteristics. A prospective cohort study of 95 patients with Crohn's disease showed that the Harvey‐Bradshaw index (HBI) score was significantly reduced and the clinical remission rate was significantly increased in patients with baseline LL‐37 levels ≥ 40 ng/mL during the 6‐ to 18‐month follow‐up period. Increased concentration of LL‐37 is also associated with an increased risk of intestinal stenosis (RR = 1.8, *p* = 0.016). These findings show that the clinical significance of LL‐37 in Crohn's disease varies with the stage of the disease, making it a dynamic biomarker that can reflect both short‐term treatment response and long‐term complication risk [74].

In summary, LL‐37 plays a context‐specific role in inflammatory bowel diseases. Its multifunctional function is dynamically regulated by the intestinal microenvironment, the specific receptor signaling pathway and the interaction with microbial components. An in‐depth understanding of these regulatory mechanisms is crucial to transform LL‐37‐related strategies into feasible clinical applications.

### 3.2 The Role of LL‐37 in Colorectal Cancer

Tumor occurrence and progression are closely related to chronic inflammation [75]. Recent studies have shown that LL‐37 has become a key regulatory factor for tumor‐related events, but its impact on different types of cancer is highly situationally dependent. Studies have confirmed that LL‐37 is up‐regulated in a variety of malignant tumors, including lung cancer, breast cancer, pancreatic cancer, prostate cancer, ovarian cancer, and malignant melanoma. Among these malignant tumors, LL‐37 is associated with tumor proliferation and invasion enhancement [40, 76, 77, 78]. On the other hand, the expression of LL‐37 is reduced in oral squamous cell carcinoma, gastric cancer, hepatocellular carcinoma, and colorectal cancer, indicating that the peptide substance can inhibit the growth of such tumors [79, 80]. However, in colorectal cancer, LL‐37 shows the dual activity of promoting tumor and anti‐tumor. These differential effects seem to depend on many variables, including peptide concentration, receptor interaction, disease stage, and tumor microenvironment (TME).

CRC is a high‐incidence malignant tumor of the gastrointestinal tract, and its incidence and mortality rate are on the rise worldwide. In addition to antibacterial effects, LL‐37 also plays a role by regulating tumor‐related signaling pathways, regulating the tumor microenvironment, and interacting with immune cells. Peptide concentration is one of the key factors affecting its activity in CRC. Experimental evidence shows that low concentrations of LL‐37 (< 10 μM) promote CRC cell proliferation, while high concentrations (> 20 μM) inhibit tumor growth [81]. At high concentrations, LL‐37 induces CRC cell apoptosis through a non‐caspase‐dependent GPCR‐p53 signaling pathway. The pathway activates and promotes the transposition of apoptosis‐inducing factor (AIF) and EndoG to the nucleus, thus inhibiting the growth of CRC cells [79]. The study also showed that LL‐37 and its synthetic analog FF/CAP‐18 can upregulate the expression of miR‐663a, resulting in a decrease in the level of CXCR4 mRNA in CRC cells and causing cell cycle blockage [82]. In addition, FF/CAP‐18 changes cell metabolism by inhibiting glycolysis and the tricarboxylic acid cycle (TCA cycle) and enhancing purine metabolism, which ultimately leads to energy depletion of HCT116 cells and induces apoptosis (10–40 μg/mL concentration range) [83]. On the contrary, low concentrations of LL‐37 may promote tumor growth. Under tumor‐like microenvironmental conditions, Pan et al. found that LL‐37 can activate the Wnt/β‐catenin signaling pathway and upregulate the expression of cell cyclin D1 and c‐Myc, thus promoting the proliferation of colorectal cancer cells [84]. These results show that the biological response of LL‐37 is inversely proportional to its local concentration, suggesting the need to establish a reasonable treatment plan based on LL‐37.

The cancer suppression and cancer promotion functions of LL‐37 in colorectal cancer are mediated by multiple receptor pathways, which activate different downstream signaling programs, respectively. These pathways are not mutually exclusive, and their relative contribution seems to depend on peptide concentration and disease stage. The study found that the activation of the GPCR‐p53 axis and purinergic receptor P2RX7 is the main mechanism of LL‐37 inhibiting tumors. The core process of its inhibitory effect is to regulate the cytoskeletal tissue. As a key component of the cytoskeleton, microtubules play a central role in cell morphology, intracellular transport, and mitosis. In CRC, class III β‐tubulin (TUBB3) is often highly expressed, which is associated with anti‐apoptosis and enhanced invasive ability [85, 86]. Wang's team confirmed that LL‐37 reduces TUBB3 expression and destroys microtubular stability by activating P2RX7, thus inhibiting CRC cell migration and invasion [87, 88]. On the contrary, the tumor‐promoting effect of LL‐37 is related to its activation of the Wnt/β‐catenin pathway and its interaction with tumor‐infiltrated immune cells. At sub‐micromolar concentrations, LL‐37 may promote the proliferation signal conduction of colorectal cancer cells. Although TLRs and FPRs are known to be the mediators of LL‐37 signaling in other situations, their role in colorectal cancer biology has not been fully clarified.

The tumor microenvironment (TME) also plays a key role in determining the biological effects of LL‐37. In addition to direct action, LL‐37 can also regulate immune cell infiltration, epithelial‐mesenchymal transformation (EMT), and the interaction between tumor cells, microorganisms, and matrix components. Therefore, the difference in the expression of LL‐37 in epithelial cells may reflect the difference in the local immune microenvironment. Porter et al. found that the expression of LL‐37 in epithelial cells was related to the progression of CRC. Its research shows that from UICC stage I to stage III, the expression of LL‐37 gradually decreased (*p* = 0.048). The downward trend has appeared in phase I compared with the normal mucosa (*p* = 0.009), and it is more significant in phases II and III (*p* < 0.001) [89]. It is worth noting that there is a significant correlation between epithelial LL‐37 expression and interstitial CD8 + T cell infiltration. These results show that LL‐37 participates in anti‐tumor immunity by promoting the recruitment or retention of cytotoxic T lymphocytes in the tumor microenvironment. EMT is a key process of invasion and metastasis of CRC. Cheng et al.'s studies confirmed that LL‐37 can inhibit the proliferation of cancer‐associated fibroblasts (CAFs) and block EMT, thus inhibiting the local invasion and metastatic spread of CRC [90]. The role of LL‐37 derived from interstitial cells in regulating the tumor microbiome is becoming a promising research direction. The latest evidence shows that transforming CAFs through optogenetic methods can achieve the in situ production of LL‐37 in breast cancer, effectively remove intratumor pathogens (*Fusobacterium nucleatum*), reshape the tumor microenvironment to block metastasis, and does not affect the systemic microbiome [91]. However, the mechanism of LL‐37 from endogenous CAF sources in CRC is unclear.

**Figure 3:**
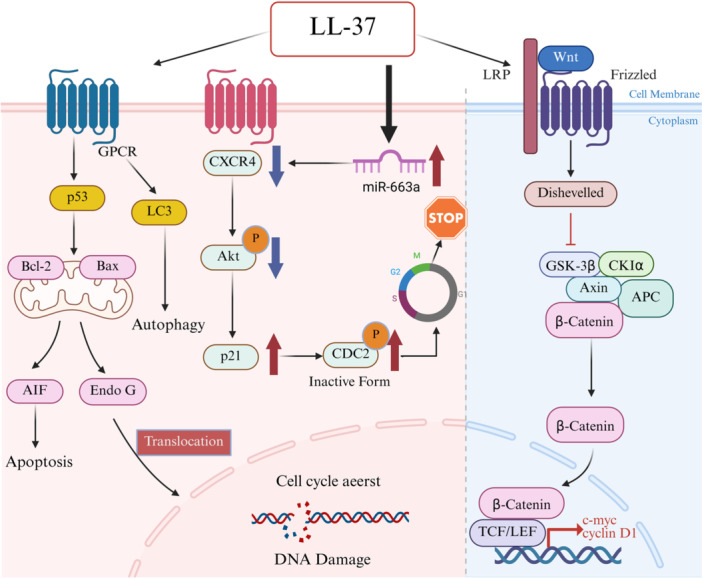
Dual Role of LL‐37 in Colorectal Cancer: Tumor Suppression and Promotion.

In summary, these findings highlight the multifunctional role of LL‐37 in CRC. Figure 3 reveals the mechanism by which LL‐37 inhibits or promotes the progression of CRC through two signaling pathways. It is worth noting that these opposite effects do not occur accidentally, but can be explained by specific identifiable factors, including peptide concentration, receptor binding status, and disease background. Table 2 summarizes the key factors driving this functional change and puts forward a conceptual framework to explain how the role of LL‐37 depends on the specific situation, which provides guidance for the development of future treatment interventions.

**Table 2:** Conditions governing the functional switch of LL‐37 between anti‐tumor and pro‐tumor activities in CRC.

Determinant	Anti‐tumor conditions	Pro‐tumor conditions	Key mechanism/Pathway
Peptide concentration	> 20 μM	< 10 μM	Biphasic dose‐response; high dose triggers apoptosis.
Receptor Specificity	GPCR‐p53 axis; P2RX7 activation	Wnt/β‐catenin activation	The differential activation of apoptotic signals and proliferative signals.
Primary Mechanism	Apoptosis induction; Cytoskeletal disruption (TUBB3 downregulation)	Facilitation of cell cycle (Cyclin D1); c‐Myc upregulation	Energy depletion and proliferation‐promoting stimulation.
TME Context	Enhanced CD8 + T cell infiltration; CAF inhibition; EMT suppression	Activation of pro‐inflammatory signaling; EMT promotion; active CAFs	Immune surveillance and regulation of matrix remodeling
Microbiome Interaction	Depletion of intratumoral pathogens	Interaction with specific bacterial metabolites	Remodeling of the “tumor microbiome”.

Abbreviations: CAFs, cancer‐associated fibroblasts; CD8 + T cell, cytotoxic T lymphocyte; CRC, colorectal cancer; EMT, epithelial‐mesenchymal transition; GPCR, G protein‐coupled receptor; P2RX7, P2X purine receptor 7; TME, tumor microenvironment; TUBB3, β 3 Tubulin.

### 3.3 The Role of LL‐37 in the Microbiome–Immune Axis and Gut Dysbiosis

#### 3.3.1 The Microbiome–Gut–Immune Axis in Intestinal Homeostasis

The gut‐immune axis is the dynamic interaction between the intestinal microbiota and the host immune system [92]. In a healthy state, this interaction promotes immune tolerance and metabolic homeostasis. Intestinal microbiota disorders may destroy the integrity of the epithelial barrier and enhance intestinal permeability. This “intestinal leakage” phenomenon causes microbial components (such as LPS) to enter the whole body circulation [93]. LPS in the blood can stimulate both congenital and adaptive immune responses. The latest study shows that LPS can induce macrophages to polarize to the M1‐type inflammatory phenotype and regulate the dynamics of T cell populations, which may lead to chronic systemic inflammation and associated neurodegenerative diseases [94].

#### 3.3.2 LL‐37 As a Key Effector of the Gut–Immune Axis

LL‐37 is an important effector molecule at the interface between the intestinal flora and the host immune system. It not only maintains the homeostatic state of microorganisms but also helps to maintain the integrity of the epithelial barrier. In healthy tissues, LL‐37 maintains the balance between symbiotic bacteria and pathogenic bacteria. Its expression is up‐regulated during infection. The main source of LL‐37 is Paneth cells in the small intestinal cavity. When invaded by microorganisms, these cells will secrete LL‐37 to provide early antibacterial defense [95]. LL‐37 participates in the regulation of the microbiome‐immune axis through multiple mechanisms: at the microbial level, it can interfere with the bacterial population sensing system (Las and Rhl) and inhibit the expression of biofilm‐related genes [96], thus preventing pathogen colonization and microbial overproliferation. In terms of mucosal defense, LL‐37 has the ability to directly destroy the microbial membrane structure. For example, LL‐37 binds to the negatively charged phosphatidylserine on the surface of the cell membrane of *Candida albicans*, forming a pore, causing the leakage of cell contents. This mechanism can also block fungal adhesion by destroying the interaction between Als3 adhesin and E‐cadherin in epithelial cells, thus limiting fungal colonization [97]. LL‐37 helps to maintain the epithelial barrier function. Marin and other researchers confirmed that LL‐37 promotes intestinal barrier repair by recombining the tightly connected structure. It can also enhance the ability of TLR4‐dependent identification of *Salmonella* LPS, thus strengthening the innate immune response and limiting the migration of bacteria through intestinal epithelial cells [98]. In addition, LL‐37 can enhance macrophage phagocytic activity in cooperation with microbial metabolites, which helps to curb the proliferation of intestinal pathogens. Through these synergistic effects, LL‐37 can reduce the severity of infectious colitis and support mucosal immune defense [99].

#### 3.3.3 Bidirectional Coupling and Neuroimmune Extensions

More and more evidence shows that there is an interaction between LL‐37 and intestinal microbiota. Under anaerobic conditions or in the presence of a large number of short‐chain fatty acids (SCFAs), symbiotic anaerobic bacteria will induce the expression of hypoxic factor‐1α (HIF‐1α), thus enhancing the expression of LL‐37 in colon epithelial cells, and then driving the transcription of the *CAMP* gene [100, 101]. Through this pathway, microbial metabolites participate in maintaining the homeostasis of the host‐microbiome by regulating the production of endogenous antimicrobial peptides. On the contrary, the low expression level of LL‐37 in the neonatal period will interfere with microbial colonization, leading to an imbalance in the microbiota and making individuals susceptible to autoimmune diseases such as type 1 diabetes [102]. It is worth noting that the regulatory effect mediated by LL‐37 is not limited to the intestine. The latest research shows that pancreatic alveolar cells secrete LL‐37 through the Orai1 calcium channel, which can affect the composition of intestinal flora and promote mucosal congenital immunity [103]. In addition, Bruzaferro and others confirmed that CRAMP maintains microbial balance by directly inhibiting microbial growth or regulating immune cell activity [104]. These findings are consistent with the existing microbiome‐immune‐neuraxial cognition and reveal that LL‐37 may be involved in neuro‐immune communication, which may have a profound impact on intestinal diseases and age‐related cognitive disorders [94].

### 3.4 Synergistic Roles of LL‐37 and Microbial Postbiotics in IBD

Restoring intestinal homeostasis of IBD is increasingly associated with strategies to regulate intestinal microbiota. Probiotics (especially *Lactobacillus* and *Bifidobacterium*) can not only inhibit the colonization of pathogens, but also promote the host to produce LL‐37 [105], thus enhancing the epithelial barrier defense function. At the same time, probiotics (short‐chain fatty acids, microbial peptides, and cell wall components), as inactive microbial products or metabolites, have attracted much attention for their enhanced stability and good safety, but they still retain the multiple immune regulation and barrier protection effects of probiotics [106, 107]. As the main energy source of colon cells in short‐chain fatty acids, butyric acid has the potential to maintain epithelial integrity by stimulating mitochondrial respiration, upregulating tight junction proteins (occludin and claudin‐1), and inhibiting NF‐κB‐mediated inflammatory signaling pathways [108]. The latest research evidence shows that probiotics and prebiotics can also enhance the host's defense ability by activating endogenous LL‐37.

LL‐37 is not only an antibacterial peptide, but also acts as a connecting medium for microbial signals and host immune response. For example, studies have shown that short‐chain fatty acids increase the expression of *CAMP* genes by inhibiting histone deacetylase (HDACs), thus enhancing the mucosal barrier function [101]. At the same time, it has been confirmed that microbial metabolites can regulate mitochondrial activity, reduce oxidative stress, and maintain epithelial integrity [109]. These effects may have a synergistic effect with the immunomodulatory characteristics of LL‐37. Therefore, the strategy of enhancing the production of LL‐37 through probiotic or prebiotic intervention can provide complementary benefits in limiting the growth of pathogens and restoring the metabolic balance of inflammatory bowel tissue.

Overall, there is growing evidence that LL‐37 plays a key role in maintaining microbial balance and coordinating intestinal antibacterial immunity. The treatment strategy to enhance the activity of LL‐37 may provide a new way to prevent flora disorders and treat infection‐related intestinal diseases.

## 4 Limitations, Optimization Strategies, and Future Prospects of LL‐37 in Intestinal Disorders

As an endogenous antimicrobial peptide, LL‐37 has broad‐spectrum biological activity, including antibacterial defense, immunomodulation, promoting tissue repair, and potential anti‐tumor effects. These multifunctional characteristics make it increasingly the focus of potential treatment targets for intestinal diseases. This section will review the main obstacles that limit the clinical application of LL‐37 at present, focus on the strategies recently developed to overcome these limitations, and explore several key directions to promote the treatment of intestinal diseases based on LL‐37 in the future.

### 4.1 The Limitations of LL‐37–Based Therapies

Although LL‐37 has diversified biological activities, the intestinal microenvironment and disease status have a profound impact on its therapeutic application. At present, there are still several factors limiting its clinical development. Cheng and other scholars pointed out a number of limitations, including cytotoxicity at high concentrations, lack of stability, protein hydrolysis degradation, and the emergence of antibiotic resistance [110].

#### 4.1.1 Cytotoxicity

When the concentration exceeds 20 μM, LL‐37 can damage red blood cells, white blood cells, and other normal tissue cells [79, 111]. Common toxic effects include hemolysis, cell membrane destruction, and apoptotic pathway activation [110, 112]. Under the background of cancer, LL‐37 shows concentration‐dependent dual functions: low concentrations can promote tumor growth, while high concentrations can inhibit tumor cells [78]. This dual effect makes it complicated to establish the best treatment window. In addition to the effect on surrounding tissues, LL‐37 is also considered to be neurotoxic. Experiments have confirmed that it can activate the CLIC1 channel, causing excessive excitation of glial cells, which may be involved in the pathogenesis of Alzheimer's disease (AD) [113]. Its pro‐inflammatory characteristics may also promote the process of neurodegenerative lesions.

#### 4.1.2 Low Stability and Metabolic Restrictions

The main challenge of therapeutic application is its low stability and low bioavailability, especially in the gastrointestinal environment. LL‐37 is extremely susceptible to protease degradation from host and pathogen origins. For example, aureolysin produced by *Staphylococcus aureus* can quickly degrade LL‐37, resulting in a significant decrease in its antibacterial activity [6, 110]. This degradation is especially obvious in the infected site with elevated protease activity. In addition, LL‐37 is cleared from the body very quickly. The peptide segment has cationic properties and is easy to bind to serum proteins, further reducing its bioavailability [13]. Therefore, the half‐life of circulating LL‐37 is usually less than an hour, and the drug needs to be given many times to reach the therapeutic concentration [114].

#### 4.1.3 Drug Resistance Challenge

More and more evidence shows that bacteria can develop adaptive resistance to LL‐37. The proposed mechanisms include: membrane charge change, efflux pump upregulation, increased degradable protease secretion, and biofilm formation enhancement [115, 116]. Experimental evolutionary studies show that *Staphylococcus aureus* can begin to reduce sensitivity in just a few generations under the action of sub‐inhibitory concentration LL‐37, and stable drug resistance can be obtained after about 168 generations [117, 118]. Similar drug resistance patterns were also observed after long‐term exposure to LL‐37 by *Salmonella typhimurium* and *Clostridium difficile* [110]. These results show that the development of therapy based on LL‐37 faces major obstacles. It is worth noting that studies have reported cross‐drug resistance between LL‐37 and polymyxin antibiotics (such as polymyxin B) [119, 120].

### 4.2 Optimization Strategies: Breaking Through the Conversion Barrier

In order to overcome the limitations of LL‐37, researchers tried to improve its therapeutic effect through peptide modification, molecular engineering, targeted delivery system development, combined therapy, and gene therapy [114]. Structural modification and analog design can enhance the stability of LL‐37 and reduce cytotoxicity. By introducing strategies such as d‐amino acid substitution, peptide cyclization, and terminal truncation, the LL‐37 variant [110, 121], which both enhances protein hydrolysis resistance and retains biological activity, has been obtained. For example, FF/CAP‐18 and its synthetic derivatives still have anti‐tumor activity, but the hemolytic toxicity is significantly reduced [122]. The delivery system based on nanotechnology can also improve the pharmacokinetic characteristics and delivery efficiency of LL‐37. Encapsulated LL‐37 in liposomes, polymer nanoparticles, or lipid carriers can protect peptides from enzymatic degradation, extend the cycle time, and achieve controlled release [123, 124]. In addition, tissue specificity and directional delivery can be achieved through surface modification of targeted ligands [125]. The magnetic nanoparticle system developed by the Wnorowska team combines LL‐37 with magnetic nanoparticles (MNPs), which significantly enhances the targeting and anti‐tumor effect of colorectal cancer cells [126]. However, the delivery system based on nanoparticles still has limitations, such as difficulties in mass production, differences between batches, and potential immunogenicity of nanocarriers [127, 128]. In order to improve the efficacy of LL‐37, the researchers also tried combined treatment strategies. The study found that the combination with traditional chemotherapy drugs can significantly inhibit the proliferation of colorectal cancer cells [129]. In addition, LL‐37 may have a synergistic effect with some antibiotics. For example, when used in combination with vancomycin, LL‐37 can enhance the antibacterial activity of multidrug‐resistant strains, indicating that it has application value in the treatment of complex intestinal infections [130].

### 4.3 Future Perspectives

Emerging evidence shows that the clinical significance of LL‐37 has gone beyond its classical antibacterial activity and should be understood under the broad framework of the intestinal‐microbiota axis. Against this background, LL‐37 not only acts as an antimicrobial peptide but also as a medium for signal transmission between microorganisms and host immune regulation. One of the new treatment ideas is to integrate LL‐37‐related mechanisms and microbiome‐targeted therapy, including prebiotics and a new generation of probiotic preparations. Such strategies do not rely on exogenous peptide delivery but induce endogenous LL‐37 generation so as to avoid potential cytotoxicity and overcome delivery problems.

SCFAs can positively regulate the expression of LL‐37, which in turn stimulates the hypoxia‐induced factor 1‐alpha (HIF‐1α) in colon epithelial cells. Therefore, enhancing the level of butyrate and other metabolites through diet or medication will promote the generation of endogenous LL‐37 and strengthen the antibacterial defense ability of the mucosa [101]. It is worth noting that the interaction between LL‐37 and microbiota is bidirectional: microbial metabolites can regulate the expression of LL‐37, and LL‐37 itself reshapes the microbial community structure by inhibiting pathogenic bacteria and stabilizing the symbiotic flora [110]. These microenvironmental changes may also promote the colonization of beneficial probiotics. In summary, these findings support a treatment model with microbiome regulation and LL‐37 regulation synergy as the core. Using this two‐way relationship, we can provide a reasonable strategy for the restoration of intestinal‐immune homeostasis in multi‐factor intestinal diseases.

## 5 Conclusion

In summary, LL‐37 is a multifunctional effector molecule in the host defense system, and its role in antibacterial defense, immune regulation, and tumor biology has been confirmed (Table 1). Clarifying the differentiated functional mechanism of LL‐37 in the physiological and pathological states is crucial for transforming the strategy based on LL‐37 into a targeted treatment of intestinal diseases.

## Author Contributions

All authors contributed to the research design. Qichao Liu and Peng Xu designed and prepared the manuscript; Cheng Zhang read and approved the final manuscript. All authors read and approved the final manuscript.

## Conflicts of Interest

The authors declare no conflicts of interest.

## Data Availability

Data sharing is not applicable to this article as no new data were created or analyzed in this study. All information discussed in this review is derived from previously published literature cited in the reference list.
